# The Spontaneous Improvement of Cricopharyngeal Achalasia in a Child with Motor Delay: A Case Report

**DOI:** 10.3390/reports8020036

**Published:** 2025-03-22

**Authors:** Marco Gitto, Anna Colombo, Alessandro Campari, Eleonora Bonaventura, Sara Rocca

**Affiliations:** 1 Department of Biomedical and Clinical Sciences, Università degli Studi di Milano, 20157 Milan, Italy; sara.rocca@unimi.it; 2 V. Buzzi Children’s Hospital, 20154 Milan, Italy; anna.colombo89@asst-fbf-sacco.it; 3 Department of Pediatric Radiology, V. Buzzi Children’s Hospital, 20154 Milan, Italy; alessandro.campari@asst-fbf-sacco.it; 4 Unit of Pediatric Neurology, Center for Diagnosis and Treatment of Leukodystrophies (COALA), V. Buzzi Children’s Hospital, 20154 Milan, Italy; eleonora.bonaventura@asst-fbf-sacco.it

**Keywords:** cricopharyngeal achalasia, motor delay, videofluoroscopic swallow study, spontaneous resolution, case report

## Abstract

**Background and Clinical Significance:** Cricopharyngeal achalasia (CPA) is a rare disorder of the upper esophageal sphincter (UES), characterized by failure of the cricopharyngeus muscle to relax during swallowing. Pediatric CPA is particularly uncommon and often associated with comorbidities, such as neurological impairments, developmental delays, and laryngomalacia. The existing literature primarily consists of small case series, limiting insights into its natural history, particularly spontaneous resolution. This case highlights a unique instance of spontaneous improvement in CPA, contributing valuable knowledge to pediatric otolaryngology and gastroenterology. **Case Presentation:** We report the case of a 38 months male with global motor delay, presenting with feeding difficulties, choking, and aspiration. A videofluoroscopic swallow study (VFSS) confirmed CPA with impaired bolus passage and posterior indentation consistent with a cricopharyngeus bar. Despite multidisciplinary consultations, interventional therapies were deferred due to parental preference and cricopharyngeal EMG findings, showing muscle inhibition during swallowing. Over six months, the patient exhibited a spontaneous resolution of feeding difficulties and aspiration, with the normalization of VFSS findings. This rare case suggests a distinct natural history of CPA in young children. **Conclusions:** This case emphasizes the role of cricopharyngeal EMG in the differential diagnosis for pediatric feeding difficulties and its potential for spontaneous resolution. It highlights the need for further research into prognostic indicators and management strategies for CPA in children, offering a hopeful perspective for clinicians and caregivers.

## 1. Introduction and Clinical Significance

Cricopharyngeal achalasia (CPA) and cricopharyngeus muscle dysfunction (CPMD) represent distinct but related disorders of the upper esophageal sphincter (UES). As documented in the literature, the cricopharyngeus muscle, which forms a posterior C-shaped band at the level of the pharyngoesophageal junction, normally maintains tonic contraction at rest and relaxes during swallowing to allow bolus passage [1]. CPA specifically refers to the failure of the cricopharyngeus muscle to relax during deglutition, while CPMD encompasses a broader spectrum of abnormalities including the failed, diminished, or uncoordinated relaxation of the UES [2].

In the pediatric population, CPA is relatively rare and can present as either primary (idiopathic) or secondary to various underlying conditions [3]. Primary CPA in children typically manifests with symptoms such as choking, regurgitation, aspiration, and failure to thrive. The condition may be associated with other pediatric disorders, including neurological conditions, laryngomalacia, and developmental delay. Notably, some cases in infants and young children have demonstrated spontaneous improvement over time, suggesting a potentially different natural history compared to adult presentations [3].

The diagnosis of pediatric CPA primarily relies on videofluoroscopic swallow studies (VFSSs), which can demonstrate a posterior indentation at the level of the cricopharyngeus muscle (CP bar) and impaired bolus passage through the UES, with the bolus typically halting above the indentation [4]. While manometry can provide objective measurements of UES pressures and function, as well as pharyngeal activity, its use in pediatric populations has been limited by technical challenges and a lack of standardized normative data [3]. Cricopharyngeal EMG allows a direct assessment of cricopharyngeal activity during swallowing; in CPA the inhibition of cricopharyngeal activity during swallowing is not expected. Additionally, EMG findings reveal abnormal cricopharyngeal muscle activity, with an altered EMG pause during swallowing [5].

Management approaches for pediatric CPA vary based on symptom severity and associated conditions. Treatment options include observation with counseling on meal consumption strategies provided by a speech and language pathologist, particularly in mild cases or when spontaneous improvement is anticipated [3]. For more severe cases, interventional options include cricopharyngeal myotomy (both open and endoscopic approaches), balloon dilation, and botulinum toxin injection. The literature suggests that botulinum toxin injection may serve as both a diagnostic tool and temporary therapeutic option, as its effects typically last between 3 and 6 months, which is particularly valuable in children where the natural history of the condition remains unclear [3].

Despite these insights, the literature on pediatric CPA remains sparse, with the majority of studies involving small cohorts or isolated case reports, as evidenced in Table 1 which summarizes key studies on pediatric CPA and their findings.

Distinguishing between CPA and CPMD, along with recognizing their unique presentations in children, enables proper diagnosis and management. This case report presents an unusual instance of spontaneous improvement in a child with CPA, contributing to the body of evidence regarding the natural history of this condition in the pediatric population.

## 2. Case Presentation

### 2.1. Case History

The patient was born at an external medical center and was subsequently referred to our outpatient phoniatric service at 16 months of age for comprehensive evaluation.

A male infant was delivered at 37 weeks and 1 day gestation via urgent cesarean section, necessitated by failure to progress and cardiotocographic abnormalities. The pregnancy was notable for third-trimester polyhydramnios. At birth, the neonate presented with cyanosis, atonia, and apnea, with bradycardia (heart rate <60 beats per minute). Apgar scores were documented as 0, 5, and 8 at 1, 3, and 5 min, respectively. Birth anthropometrics included weight of 2610 g (25th percentile), length of 49 cm (35th percentile), and head circumference of 36 cm (75th percentile).

The immediate postnatal period required therapeutic hypothermia protocol implementation for 72 h due to hypoxic-ischemic encephalopathy. The patient necessitated non-invasive ventilation with high flows for 1 month. Initial neurological examination revealed impaired sucking reflexes. Comprehensive genetic investigations, including Array CGH and targeted genetic panel analysis for encephalopathies and epilepsies, yielded negative results.

### 2.2. Neurological Evolution and Developmental Trajectory

Further neurological assessment revealed a consistent pattern of global developmental delay (June 2023, age 1 year, 8 months). The patient’s motor function showed initial hypotonia that progressed to variable muscle tone over time. Oral-motor coordination demonstrated persistent impairment of voluntary movement patterns. The progression of developmental milestones showed significant delays, with the patient achieving independent sitting and crawling at 11 months, supported forward walking at 13 months, and limited independent forward walking at 15 months. Communication development was notably delayed, with expressive language limited to isolated words (October 2023, age 2 years).

### 2.3. Swallowing Dysfunction and Diagnostic Progression

#### 2.3.1. Early Manifestations

Initial feeding difficulties presented as a complex syndrome characterized by impaired sucking–swallowing coordination, excessive oral secretion management difficulties, recurrent choking, and clinical features consistent with neurogenic dysphagia, evaluated during neurological examination (October 2023, age 1 year) and VFSS (June 2022, age 8 months; June 2023, age 1 years, 8 months).

#### 2.3.2. Sequential Diagnostic Evaluations

The initial VFSS (June 2022, age 8 months) demonstrated significant swallowing mechanism dysfunction. Findings included prolonged initiation phase of swallowing reflex, impaired UES relaxation, silent aspiration events, altered bolus transit dynamics, and evidence of cricopharyngeal dysfunction.

Direct Laryngoscopic Evaluation (March 2023, age 1 year, 4 months) revealed residual material accumulation in the oropharyngeal complex, while demonstrating preserved laryngeal mobility patterns and normal vocal fold function; no evidence of posterior shelf at the level of the UES was found. The patient showed toleration of homogeneous semi-solid consistencies, with no structural abnormalities identified.

**Figure 1:**
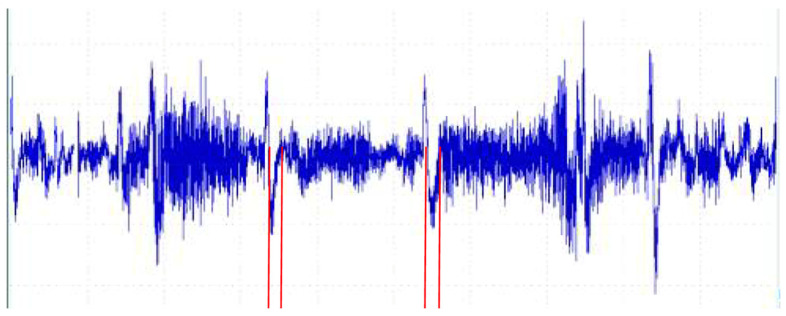
EMG findings of left cricopharyngeus muscle during swallowing. Graph shows recordings of electrical activity with needle electrodes, highlighting both baseline muscle tone and activity patterns during swallowing. Waveform demonstrates consistent inhibition of cricopharyngeal muscle activity during spontaneous swallowing, evidenced by red lines that represent action potentials, with duration of approximately 150 ms per event.

Follow-up VFSS (June 2023, age 1 year, 8 months) documented progression of swallowing dysfunction, characterized by prolonged oral and pharyngeal phase duration, persistent pharyngeal residue, and nasopharyngeal regurgitation. Silent aspiration episodes persisted, and progressive cricopharyngeal thickening development was noted (Figure 1).

**Figure 2:**
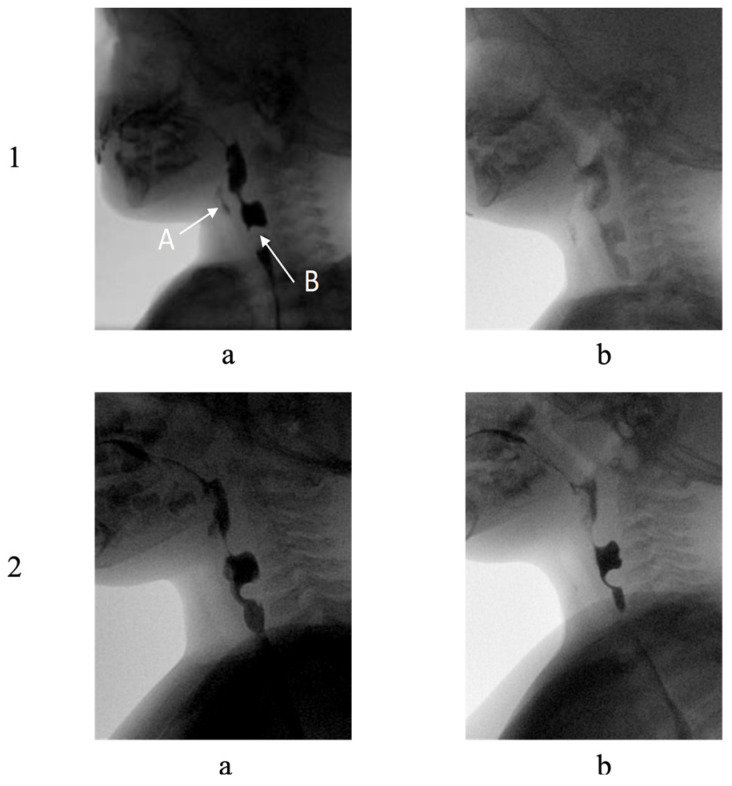
Lateral-view frames from VFSS performed in June 2023 (row 1) and April 2024 (row 2) with depiction of cricopharyngeus bar during bolus transit through oropharynx. In row 1, Panels (a) and (b) illustrate swallowing of two distinct consistencies: (a) liquid (water combined with contrast medium) and (b) solid (snack with contrast). In panel (a), A illustrates laryngeal aspiration and B illustrates cricopharyngeus bar. In row 1, Panels a and b depict swallowing of (a) semisolid (contrast-enhanced pudding) and (b) soft solid (contrast-enhanced snack).

Electromyographic (EMG) analysis (March 2024, age 2 years, 4 months) demonstrated baseline cricopharyngeal muscle tone within normal limits, with a preserved inhibitory pause duration of 150 milliseconds (Figure 2). The timing of muscle relaxation was appropriate, with normal activation–relaxation patterns.

The final VFSS (May 2024: age 2 years, 6 months) evidenced significant functional improvement. The examination showed mild oral phase prolongation with bolus fragmentation and compensatory lingual hypermotility. Pharyngeal phase coordination had improved, without aspiration events noted. Significant penetration had resolved, and despite the persistent cricopharyngeal bar, adequate bolus passage was observed.

The progression from the second VFSS exam to the final VFSS exam is illustrated in Figure 1.

#### 2.3.3. Therapeutic Management

The management strategy encompassed three primary domains. Nutritional support included percutaneous endoscopic gastrostomy placement (February 2022, age 3 months) and Nissen fundoplication for reflux management (March 2022, age 4 months). The current nutritional regimen consists of *Nestlè Compleat* formula (1.2 kcal/mL) administered as three daily boluses of 250 mL, totaling 900 kcal daily, with regular nutritional status monitoring. The rehabilitation program incorporated structured swallowing therapy, progressive oral intake trials, regular functional reassessment, and implementation of compensatory strategies. Multidisciplinary care coordination included integrated speech and language therapy, specialized feeding therapy, ongoing neurological monitoring, regular developmental assessment, and respiratory status surveillance.

#### 2.3.4. Additional Diagnostic Findings

Brain Magnetic resonance imaging (MRI) (January 2024, age 2 years, 2 months) revealed significant neurological findings: microcephalic tendency, corpus callosum thinning, ventricular system prominence, and subarachnoid space enlargement. Previously documented subdural hematomas (October 2021) had resolved. Laboratory analysis (March 2024, age 2 years, 4 months) showed adequate vitamin D levels (45.9 mcg/L), elevated hemoglobin A2 fraction, and normal hemoglobin F fraction. Both sweat test and celiac disease screening returned negative results. The longitudinal evaluation of this case demonstrates a pattern of spontaneous improvement in swallowing function, particularly evident in the tendency for normalization of cricopharyngeal muscle function, as documented through serial instrumental assessments.

#### 2.3.5. Timeline

The longitudinal progression of the patient’s condition, from birth to early childhood, is documented chronologically in Table 2. This timeline captures key diagnostic evaluations and therapeutic interventions, illustrating the temporal relationship between cricopharyngeal dysfunction improvement and developmental milestones.

## 3. Discussion

This case presents several aspects regarding the spontaneous improvement of CPA in a pediatric patient with complex comorbidities. The potential role of cricopharyngeal EMG is also relevant. The documented natural improvement contributes significantly to the understanding of conservative management approaches in pediatric CPA, particularly given the limited longitudinal data available in the current literature [1].

Current therapeutic options for pediatric CPA span a spectrum from conservative management to invasive interventions [3]. Treatment modalities include botulinum toxin injection, endoscopic dilation, and surgical myotomy, with reported success rates varying significantly. Botulinum toxin injection demonstrates variable outcomes, with success rates ranging from 43% to 100% in pediatric populations [3]. While endoscopic dilation offers a less invasive approach than myotomy, it frequently necessitates repeated interventions and carries inherent risks of perforation and mediastinitis [2]. Surgical myotomy, though considered a definitive treatment with success rates of 75–90%, involves significant operative risks that warrant careful patient selection [3].

The spontaneous improvement observed in our case, documented through sequential diagnostic studies, represents a particularly significant finding. Our diagnostic approach, utilizing serial videofluoroscopic studies and EMG, aligns with validated assessment protocols described in recent multicenter studies [4].

This management approach suggests the possibility of non-invasive interventions, supported by emerging evidence favoring conservative initial management in selected cases [3], and aligns with the current literature suggesting that expectant management may be appropriate in cases showing progressive improvement [1].

The temporal relationship between developmental progression and swallowing improvement raises questions about neuroplasticity in pediatric CPA recovery. This observation is consistent with the documented cases in recent systematic reviews highlighting the potential for spontaneous improvement in pediatric populations [3]. The parallel improvement in motor milestones and swallowing function suggests potential mechanistic links between general motor development and cricopharyngeal function in the developing nervous system.

Several limitations must be acknowledged. The natural history of pediatric CPA remains poorly understood, with limited longitudinal data available [2]. The presence of multiple comorbidities makes it challenging to isolate specific factors contributing to both the initial dysfunction and subsequent improvement, a common limitation noted in pediatric CPA literature [3].

## 4. Conclusions

This case report documents the spontaneous improvement of CPA in a pediatric patient with complex comorbidities, including developmental delay. The progression from severe initial dysfunction to normalized cricopharyngeal muscle function, objectively documented through sequential diagnostic studies, provides insights into the natural history of pediatric CPA.

Key findings emerge from this case. First, the spontaneous improvement of CPA is possible even in the presence of significant comorbidities, suggesting that conservative management may be appropriate in selected pediatric cases. Second, serial objective evaluations using videofluoroscopy and electromyography provide important data for monitoring disease progression and guiding therapeutic decision-making.

These observations have important implications for clinical practice. While various interventional options exist for CPA management, including botulinum toxin injection, dilation, and myotomy, this case supports a graduated therapeutic approach beginning with conservative measures in appropriately selected patients. The documented spontaneous improvement challenges the traditional paradigm of early surgical intervention and emphasizes the importance of careful patient selection for invasive procedures.

Future research should focus on identifying predictive factors for spontaneous improvement and developing standardized protocols for monitoring disease progression. Prospective studies examining the natural history of pediatric CPA, particularly in the context of developmental delay and other comorbidities, would further enhance the understanding of this condition and optimize patient-specific treatment strategies.

## Figures and Tables

**Table 1:** Studies on pediatric CPA and main findings.

First Name	Year	No. of Patients	Primary/ Associated	Diagnostics	Treatment
Basler [6]	2019	2	Primary, Associated	VFSS	Endoscopic laser-assisted CPM
				Gastrointestinal endoscopy	
				Esophageal manometry	
				pH probe monitoring	
Gollu [7]	2016	30	Primary, Associated	VFSS	Dilatation, swallowing therapy
				24 h pH probe monitoring	
				Upper gastrointestinal endoscopy	
Erdeve [8]	2007	1	Primary	Chest X-ray	Balloon dilatation
				Barium swallow	
				Manometry	
				Bronchoscopy	
				Esophagoscopy	
Scholes [9]	2014	6	Primary, Associated	Fluoroscopy studies, including video swallow studies and esophagrams (used in all patients)	Botulinum toxin injections, CPM
				Manometry and impedance studies (used in 4 of 6 patients)	
				Brain MRI (used in 3 of 6 patients)	
				Endoscopy (used in some patients)	
Drendel [10]	2013	4	Primary, Associated	VFSS	Botulinum toxin injections, CPM, balloon dilatation
				Head and neck MRI	
				Electroencephalogram	
Givens [11]	2018	1	Associated	Flexible fiberoptic laryngoscopy	Botulinum toxin injections
				FEES	
				VFSS	
Sewell [12]	2005	1	Primary	Oropharyngeal motility study	Botulinum toxin injections, CPM
				Esophagoscopy	
				Head CT	
				Brain and brainstem MRI	
Korakaki [13]	2004	1	Primary	Barium swallow	CPM
				Esophagoscopy	
				Laryngoscopy	
				Bronchoscopy	
				MRI	
Muraji [14]	2002	4	Primary	Esophagography/upper gastrointestinal series	CPM
				Manometry	
				Cine-fluoroscopic/ VFSS	

Notes: Videofluoroscopic swallow study (VFSS), cricopharyngeal myotomy (CPM), magnetic resonance imaging (MRI), and functional endoscopic evaluation of swallowing (FEES).

**Table 2:** Chronological summary of clinical events and examinations.

Date	Age	Event/Examination	Significant Findings
13 October 2021	Birth	Delivery	Urgent cesarean section at 37 weeks and 1 day due to failure to progress and CTG abnormalities. Perinatal asphyxia: cyanotic, atonic, and apneic at birth. Apgar scores: 0 (1′), 5 (3′), and 8 (5′). Weight 2610 g, length 49 cm, and HC 36 cm. Therapeutic hypothermia initiated for hypoxic-ischemic encephalopathy.
13 February 2022	3 months	PEG placement	Performed for management of dysphagia and feeding difficulties.
March 2022	4 months	Nissen fundoplication	Performed for reflux management.
June 2022	8 months	Initial VFSS	Demonstrated significant swallowing mechanism dysfunction, impaired UES relaxation, and silent aspiration events.
March 2023	1 year, 4 months	Direct laryngoscopic evaluation	Revealed residual material accumulation in oropharyngeal complex, preserved laryngeal mobility patterns, and normal vocal fold function.
June 2023	1 year, 8 months	Follow-up VFSS	Documented progression of swallowing dysfunction, prolonged oral and pharyngeal phase, persistent pharyngeal residue, nasopharyngeal regurgitation, silent aspiration, and progressive cricopharyngeal thickening.
January 2024	2 years, 2 months	Brain MRI	Revealed microcephalic tendency, corpus callosum thinning, ventricular system prominence, and subarachnoid space enlargement. Previously documented subdural hematomas had resolved.
March 2024	2 years, 4 months	EMG analysis	Normal parameters with preserved inhibitory pause duration (150 ms), appropriate timing of muscle relaxation, and normal activation–relaxation patterns.
April 2024	2 years, 5 months	VFSS	Showed mild oral phase prolongation with bolus fragmentation, improved pharyngeal phase coordination, minimal aspiration events, and adequate bolus passage despite persistent cricopharyngeal bar.
May 2024	2 years, 6 months	Final VFSS	Confirmed continued improvement in swallowing function with only minimal residual findings.

Notes: Videofluoroscopic swallow study (VFSS); percutaneous endoscopic gastrostomy (PEG); cardiotocography (CTG); head circumference (HC); upper esophageal sphincter (UES); electromyography (EMG); magnetic resonance imaging (MRI); and computed tomography (CT).

## Data Availability

Our data are unavaible due to privacy restriction. An anonymous and edited version would be provided on request.
